# AGEISM SOURCES AFFECT TECHNOLOGY ACCEPTANCE ON WEARABLE ROBOTS: A RANDOMIZED BETWEEN-SUBJECT EXPERIMENT

**DOI:** 10.1093/geroni/igae098.4270

**Published:** 2024-12-31

**Authors:** Clio Yuen Man Cheng, Vivian Weiqun Lou

**Affiliations:** The University of Hong Kong, Hong Kong, China (People’s Republic); The University of Hong Kong, Hong Kong, China (People’s Republic)

## Abstract

Existing literature shows that ageism contributes to the widening of digital divide. However, it remains largely mysterious how ageism specifically impacts technology acceptance, particularly in the context of soft wearable robots. This study aims to fill this gap by conducting a randomized between-subject experiment with six animation vignettes: self-directed ageism, ageism from family and friends, ageism from technology developers, ageism from strangers, unframed, and control. A total of 237 older adults aged 50 to 74 in Hong Kong joined the experiment. The Kruskal-Wallis tests show group significant difference in the intention to use wearable robots, χ2(5) = 12.470, p =.029. Specifically, the study found that ageism from technology developers had a significant negative impact on the intention to use wearable robots, in contrast to ageism from family and friends or strangers. Additionally, ageism from family and friends was associated with a significantly higher perceived external control of wearable robots, χ2(5) = 12.184, p =.032, as well as a significantly higher perceived performance of wearable robots, χ2(5) = 14.767, p =.011, when compared to ageism from technology developers and the control condition. Ageism from family and friends yielded a notably higher perception of health benefits associated with wearable robots, χ2(5) = 23.766, p <.001, surpassing self-directed ageism, ageism from technology developers, and the control condition. These findings highlight the distinct effects of ageism from different sources on wearable robot acceptance, underscoring the importance of considering the specific origins of ageism when examining its impact on technology acceptance.

